# Detecting associated genes for complex traits shared across East Asian and European populations under the framework of composite null hypothesis testing

**DOI:** 10.1186/s12967-022-03637-8

**Published:** 2022-09-23

**Authors:** Jiahao Qiao, Zhonghe Shao, Yuxuan Wu, Ping Zeng, Ting Wang

**Affiliations:** 1 Department of Biostatistics, School of Public Health, Xuzhou Medical University, Xuzhou, 221004 Jiangsu China; 2 Center for Medical Statistics and Data Analysis, Xuzhou Medical University, Xuzhou, 221004 Jiangsu China; 3 Key Laboratory of Human Genetics and Environmental Medicine, Xuzhou Medical University, Xuzhou, 221004 Jiangsu China; 4 Key Laboratory of Environment and Health, Xuzhou Medical University, Xuzhou, 221004 Jiangsu China; 5 Engineering Research Innovation Center of Biological Data Mining and Healthcare Transformation, Xuzhou Medical University, Xuzhou, 221004 Jiangsu China

**Keywords:** Trans-ethnic genetic overlap, Composite null hypothesis testing, Summary statistics, Gene-centric association analysis, CONTO, Joint significance test, Genome-wide association study

## Abstract

**Background:**

Detecting trans-ethnic common associated genetic loci can offer important insights into shared genetic components underlying complex diseases/traits across diverse continental populations. However, effective statistical methods for such a goal are currently lacking.

**Methods:**

By leveraging summary statistics available from global-scale genome-wide association studies, we herein proposed a novel genetic overlap detection method called CONTO (COmposite Null hypothesis test for Trans-ethnic genetic Overlap) from the perspective of high-dimensional composite null hypothesis testing. Unlike previous studies which generally analyzed individual genetic variants, CONTO is a gene-centric method which focuses on a set of genetic variants located within a gene simultaneously and assesses their joint significance with the trait of interest. By borrowing the similar principle of joint significance test (JST), CONTO takes the maximum *P* value of multiple associations as the significance measurement.

**Results:**

Compared to JST which is often overly conservative, CONTO is improved in two aspects, including the construction of three-component mixture null distribution and the adjustment of trans-ethnic genetic correlation. Consequently, CONTO corrects the conservativeness of JST with well-calibrated *P* values and is much more powerful validated by extensive simulation studies. We applied CONTO to discover common associated genes for 31 complex diseases/traits between the East Asian and European populations, and identified many shared trait-associated genes that had otherwise been missed by JST. We further revealed that population-common genes were generally more evolutionarily conserved than population-specific or null ones.

**Conclusion:**

Overall, CONTO represents a powerful method for detecting common associated genes across diverse ancestral groups; our results provide important implications on the transferability of GWAS discoveries in one population to others.

**Supplementary Information:**

The online version contains supplementary material available at 10.1186/s12967-022-03637-8.

## Background

The past decade has witnessed great success of global-scale genome-wide association studies (GWASs) in discovering a large number of single nucleotide polymorphisms (SNPs) associated with many diseases and traits in diverse ancestries (e.g., blood lipids [1], prostate cancer [2], blood pressure [3, 4], type II diabetes [5, 6], glycemic traits [7], and schizophrenia [8]). These discoveries dramatically revolutionize our biological knowledge regarding genetic basis underlying complex phenotypes [9–11], and pave the way toward novel molecular therapeutic strategies for complex diseases and disorders [12–16]. One of the important findings of trans-ethnic GWASs is that a few of association signals identified in one population are found to be replicated in others in the sense that trait-associated genes exhibit consistently statistical association and effect direction in multiple populations [12, 14, 17–21], indicating that the same trait shares similar genetic components across diverse continental populations [22–28].

Indeed, current GWASs have sought to identify trait-associated genetic loci in the hope that discoveries in one population are likely transferred to others. However, given the population genetic differentiation among various ancestral groups worldwide [14, 29–31], the extent to which discovered associations can be generalized across populations is not completely known. We herein aim to formally investigate a central problem in population genetics using widespread summary statistics data publicly available from large-scale GWASs in different populations: are significant genes associated with a complex trait in one population also statistically related to the same trait in another population?

Understanding of shared genetic foundation for traits across diverse populations has profound implications from both statistical and practical perspectives. First, it helps improve power for trans-ethnic analysis by integrating multiple studies from various ethnicities [11, 32–35], increase accuracy of trans-ethnic genetic risk prediction in racial and ethnic minorities [28, 36], and enhance resolution in fine-mapping causal genes in various populations [37, 38]. Second, it offers additional insight into biological mechanism underlying complex diseases and helps measure the extent of interaction between genetic and environmental influences on disease risk [14]. Third, it holds the key to examine the transferability of associations discovered from current medical genomics researches which focus primarily on European (EUR) descent to other populations; that is, it is greatly of interest to examine whether the identified associations also hold in other populations and even discover more trait-related loci with higher power by leveraging genetic similarity across populations [39–41]. Fourth, biologically, replicating trait-associated genes across various ancestral groups is crucial for identifying truly causal genes as genetic loci that are simultaneously related to the trait in various populations much more likely contain important causal variants.

To assess the common genetic component underlying traits across distinct populations, novel trans-ethnic genetic correlation has been proposed using only summary statistics data [26, 28, 42]. Conceptually, trans-ethnic genetic correlation quantifies the extent to which a set of SNPs exert the same or similar effects on phenotypic variation in various ancestral groups. Although such correlation provides an overall insight into genetic foundation of the trait shared between populations, it cannot characterize detailed association pattern for individual genetic loci or genes. Moreover, a near-zero estimate of trans-ethnic genetic correlation does not necessarily indicate the absence of genetic overlap because mixed trans-ethnic genetic correlations in both positive and negative directions might dilute the overall estimate. The standard GWAS conventionally considers only significant genetic loci to examine whether they could be consistently discovered across ethnicities [19], which however ignores many significantly insignificant ones but with weak effects and thus might lead to biased conclusions. In addition, trans-ethnic meta-analysis is widely used [11, 32–35], whereas the determined associations might be present only in a single population. To our knowledge, only few statistical methods have been currently developed to identify trans-ethnic trait-associated genes shared across the entire genome.

To fill this knowledge gap, in the present study we propose a novel gene-centric genetic overlap detection method called CONTO. Unlike previous studies which analyzed individual SNPs [19, 28, 43], we instead focus on a set of SNPs located within a gene simultaneously and assess their joint significance with the trait of interest. From a statistical perspective, we observe that the identification of population-common genes across the whole genome can be effectively handled under the high-dimensional framework of composite null hypothesis testing by borrowing the idea of joint significance test (JST). Methodologically, JST employs the maximum *P* value of multiple associations as the significance measurement [44] and can be equivalently expressed as a combination of three disjoint component null hypotheses [45]. However, JST is often overly conservative because it depends on the 0–1 uniform distribution as its null distribution, which fails to consider the nature of composite null hypothesis test [46].

We make two key improvements of CONTO relative to JST. First, it constructs three-component mixture null distribution by taking the nature of composite null hypothesis test into account [47]. Second, it generates decorrelated test statistics to explain the trans-ethnic genetic correlation, which ultimately leads to well-calibrated *P* value for significance evaluation. Consequently, CONTO corrects the conservativeness of JST and is expected to more powerful than JST, which is validated through a wide range of simulation scenarios. We finally applied CONTO to detect population-common genes for 31 complex traits between the East Asian (EAS) and EUR populations. We identified many shared trait-associated genes that had otherwise been missed by JST. We also revealed that population-common genes were generally more evolutionarily conserved than population-specific ones.

## Methods

### Gene-set association method

Let the marginal *Z* score and *P* value of a gene for the analyzed trait in the EAS and EUR populations to be *Z*_1_ and *Z*_2_, and *P*_1_ and *P*_2_, respectively. These gene-level summary statistics can be easily to obtain with SNP-level association results publicly released by GWASs [48–51]. Therefore, we first aggregate multiple association signals at the SNP level into a single association signal at the gene level [52]. To this aim, we employ a powerful gene-set based association method called MAGMA [53], which is efficiently conducted via user-friendly software. Afterwards, the *P* value for each gene is obtained in both populations, which is immediately converted into *Z* score. The direction of *Z* score is determined by the sign of the summation of the product of effect sizes and MAFs across all SNPs of that gene [54, 55]. These gene-level summary statistics would be taken as inputs to measure the evidence of gene association with the trait in the two populations.

### Trans-ethnic genetic overlap test under the composite null hypothesis framework

Our primary objective is to examine whether a particular trait-associated gene identified in one population is also significant in another population throughout the entire genome. The trans-ethnic genetic overlap can be defined in terms of distinct types of summary statistics. For example, the alternative hypothesis implies that both |*Z*_1_| and |*Z*_2_| are larger than a pre-assigned threshold value or that both *P*_1_ and *P*_2_ are less than a given significance level [say *α*; in our analysis we sought to control false discovery rate (FDR)]. This alternative hypothesis corresponds to three null hypotheses: (i) *H*_00_: the gene is not associated with the trait in either population; (ii) *H*_10_: the gene is associated with the trait in the first population but not the second; (iii) *H*_01_: the gene is associated with the trait in the second population but not the first. Formally, if defining the hypothesis test according to *P* values, we have

$$ H_{{0}} \; = \;\left\{ \begin{gathered} H_{01} :\;\;P_{1} \; > \;\alpha \;{\text{and}}\;P_{2} \; \le \;\alpha \hfill \\ H_{10} :\;\;P_{1} \; \le \;\alpha \;{\text{and}}\;P_{2} \; > \;\alpha \hfill \\ H_{00} :\;\;P_{1} \; > \;\alpha \;{\text{and}}\;P_{2} \; > \;\alpha \hfill \\ \end{gathered} \right.\;\;{\text{vs}}.\;\;H_{{1}} \; = \;H_{{{11}}} :\;P_{1} \; \le \;\alpha \;{\text{and}}\;P_{2} \; \le \;\alpha $$

Under this framework we intend to identify shared trait-associated genes in both populations from the viewpoint of composite null hypothesis testing.

### CONTO: composite null hypothesis test for trans-ethnic genetic overlap

Like JST, we take *P*_max_ = max (*P*_1_, *P*_2_) as our test statistic for the detection of trans-ethnic genetic overlap. However, in contrast to JST which uses the 0–1 uniform distribution as its null distribution, we directly build the null distribution of *P*_max_ to correct the conservativeness of JST by borrowing the idea given in [47], which was proposed under the context of high-dimensional epigenetic mediation analysis [45]. Specifically, we estimate the proportions of the three sub-null hypotheses and fit a mixture null distribution for *P*_max_

1 $$ \begin{aligned} \Pr (P_{{{\text{max}}}} \; \le \;\alpha |H_{0} )\; = \; & \Pr (P_{1} \; \le \;\alpha |H_{01} )\Pr (P_{2} \; \le \;\alpha |H_{01} )\Pr (H_{01} ) \\ + \; & \Pr (P_{1} \; \le \;\alpha |H_{10} )\Pr (P_{2} \; \le \;\alpha |H_{10} )\Pr (H_{10} ) \\ + \; & \Pr (P_{1} \; \le \;\alpha |H_{00} )\Pr (P_{2} \; \le \;\alpha |H_{00} )\Pr (H_{00} ) \\ \; = \; & {\uplambda }_{01} p_{01} \alpha \; + \;{\uplambda }_{10} p_{10} \alpha \; + \;{\uplambda }_{00} \alpha^{2} \\ p_{01} \; = \; & \Pr (P_{2} \; \le \;\alpha |H_{01} ) \\ p_{10} \; = \; & \Pr (P_{1} \; \le \;\alpha |H_{10} ) \\ \end{aligned} $$

where *p*_01_ is the power of rejecting *P*_2_ ≤ *α* under *H*_01_ and *p*_10_ is the power of rejecting *P*_1_ ≤ *α* under *H*_10_, both of which are estimated via the Grenander method [56]; λ_01_, λ_10_, and λ_00_ are the proportions for the three sub-null null hypotheses, all of which are calculated with well-established methods for estimating FDR [57, 58]; see more details in Additional file 1.

It needs to highlight that the proposed method above implicitly assumes that the two *P* values are uncorrelated with each other. Although this condition is guaranteed by the sequential negligibility assumption in the mediation analysis [45, 59, 60], such independence does not necessarily hold in trans-ethnic genetic overlap test because of pervasive cross-population genetic correlation [26, 28, 42], which could cause inflated false discoveries than expected if not properly handled. Therefore, when implementing our method, we first decorrelate test statistics for each gene across populations by multiplying *Z* scores by the inverse of a correlation matrix. The cross-population correlation coefficient is calculated with *Z* scores of null genes (e.g., those with *P*_1_ > 0.05 and *P*_2_ > 0.05) [61, 62]. The uncorrelated *Z* scores can be in turn transformed into two-sided *P* values based on the normal approximation. Theoretically, this decorrelation strategy maximizes the transformed test statistics and the original ones [63]; therefore, it has the minimal influence on identifying shared associations. We refer to the above method as CONTO. The code for implementing CONTO is freely available at https://github.com/biostatpzeng/CONTO.

### Simulation studies and real data applications

#### Simulation settings

We here implemented simulation studies to evaluate the performance of CONTO. Because it is conducted with only summary-level data, we thus directly sampled two sets of *Z* scores from a given multivariate normal (MVN) distribution under various scenarios. Specifically, for a gene in the first population, we generated its *Z* scores randomly from MVN((0, 0), Λ) under *H*_00_ with a probability λ_00_, or from MVN((*τ*_10_, 0), Λ) under *H*_10_ with a probability λ_10_, or from MVN((*τ*_01_, 0), Λ) under *H*_01_ with a probability λ_01_, or from MVN((*τ*_11_, 0), Λ) under *H*_11_ with a probability λ_11_. For the same gene in the second population, we drew its *Z* scores at random from MVN((0, 0), Λ) under *H*_00_ with a probability π_00_, or from MVN((0, 0), Λ) under *H*_10_ with a probability π_10_, or from MVN((0, *τ*_01_), Λ) under *H*_01_ with a probability λ_01_, or from MVN((0, *τ*_11_), Λ) under *H*_11_ with a probability λ_11_. The magnitude of *τ*_10_ (or *τ*_01_ and *τ*_11_) measures the strength of association, with larger value indicating stronger association signal. For simplicity, we set *τ*_10_ = *τ*_01_ = *τ*_11_ = 2, 3 or 4, and the total number of genes to 10000, 15000, or 20000.

After obtaining *Z* scores, we transformed them into *P* values based on the normal approximation. In our simulation, we set Λ to be a two-dimensional identify matrix. We considered three various probability settings with λ_11_ ≠ 0 to evaluate FDR control and power: (i) λ_00_ = 0.40, λ_10_ = 0.20, λ_01_ = 0.20, and λ_11_ = 0.20, constructing a highly polygenic but less overlapped genetic architecture, in which 40% genes were related to the trait in each population. and approximately 33.3% of associated genes were shared across populations; (ii) λ_00_ = 0.80, λ_10_ = 0.05, λ_01_ = 0.05, and λ_11_ = 0.10, building a less polygenic and moderately overlapped genetic architecture, in which 15% genes were related to the trait in each population and approximately 50% of associated genes were shared across populations; (iii) λ_00_ = 0.90, λ_10_ = 0.01, λ_01_ = 0.01, and λ_11_ = 0.08, generating a sparse but highly overlapped genetic architecture, in which 9% genes were related to the trait in each population, but approximately 80% of associated genes were shared by the trait across populations. Note that, to a great extent, these simulation parameters were selected based on our results of real data applications (see below).

Besides CONTO, for comparison we also carried out three other composite null methods (Additional file 1), including JST [44], joint significance composite-null test (JT-comp) [64], and divide-aggregate composite-null test (DACT) [65]. We repeated 10^3^ times for each simulation setting and displayed the average across these replicates for these methods.

#### Summary statistics of 31 complex diseases from the EAS and EUR populations

We applied these methods to 31 complex traits of EAS-only or EUR-only individuals available from distinct GWAS consortia (Table 1 and Additional file 1: Table S1). These traits were analyzed in our previous work and more detailed descriptions regarding them can be found therein and in respective original paper [28, 62]. We downloaded summary statistics of these traits and performed stringent quality control in both populations for each trait: (i) removed SNPs without rs label; (ii) filtered out non-biallelic SNPs and those with strand-ambiguous alleles; (iii) deleted SNPs whose alleles did not match with those in the 1000 Genomes Project [66]; (iv) excluded duplicated SNPs and those with inconsistent alleles between EAS and EUR populations; (v) kept only common SNPs (MAF  > 1%) which were shared in the two populations; (vi) removed SNPs located within the major histocompatibility complex region because of its complicated LD structure.

**Table 1:** Number of associated SNPs discovered by JST and CONTO for traits in the EAS and EUR populations

trait	JST (*f*_11_)	CONTO			trait	JST (*f*_11_)	CONTO		
		*f*_10_	*f*_01_	*f*_11_			*f*_10_	*f*_01_	*f*_11_
SCZ	21	0	186	57	eGFR	71	22	205	312
RA	27	5	48	87	ANM	28	7	100	127
T2D	293	115	310	824	PLT	261	29	429	625
COA	19	14	112	111	RBC	195	8	568	438
AOA	22	19	57	53	MCV	351	30	388	782
PCA	27	1	44	85	HCT	40	3	315	281
BMI	95	1	1027	291	MCH	251	20	316	726
Height	698	228	455	1544	MCHC	136	23	125	224
DBP	33	0	802	130	HGB	34	7	303	182
SBP	84	4	643	252	MONO	40	4	250	151
PP	57	2	315	122	NEUT	44	0	158	135
HDL	113	52	0	305	EO	40	2	259	173
LDL	74	22	18	166	BASO	32	5	29	101
TC	101	64	22	204	LYMPH	29	1	163	78
TG	42	41	14	109	WBC	69	12	149	259
HbA1c	56	47	34	96					

*f*_10_ and *f*_01_ are the number of identified genes that were only associated with the trait in the EAS or EUR population, respectively, and *f*_11_ is the number of shared associated genes in both populations

*SCZ* schizophrenia, *RA* rheumatoid arthritis, *T2D* type 2 diabetes, *COA* childhood-onset asthma, *AOA* adult-onset asthma, *PCA* prostate cancer, *BMI* body mass index, *DBP* diastolic blood pressure, *SBP* systolic blood pressure, *PP* pulse pressure, *HDL* high density lipoprotein cholesterol, *LDL* low density lipoprotein cholesterol, *TC* total cholesterol, *TG* triglyceride, *HbA1c* hemoglobin A1c, *eGFR* estimated glomerular filtration rate, *ANM* age at natural (non-surgical) menopause, *PLT* platelet count, *RBC* red blood cell count, *MVC* mean corpuscular volume, *HCT* hematocrit, *MCH* mean corpuscular hemoglobin, *MCHC* mean corpuscular hemoglobin concentration, *BASO* basophil count, *LYMPH* lymphocyte count, *WBC* white blood cell count

After quality control, we implemented MAGMA with genotypes of 504 EAS or 503 EUR individuals from the 1000 Genomes Project as the reference panel. We defined the set of cis-SNPs for a specific gene in terms of the annotation file provided by VIGAS [67]. The *P* value and *Z* score for each gene of traits were thus available. To handle possible residual influence of population stratification, family structures and cryptic relatedness [68–71], we further conducted genomic control for the gene-based association results of MAGMA if an inflation in these gene-level test statistics was observed (indicated by the inflation factor  > 1.05). We took the resulting *P* values or *Z* scores as input to implement JST, JT-comp, DACT and CONTO for detecting trait-associated genes shared across the EAS and EUR populations.

Afterwards, for each trait we could classify these genes into three groups: (i) null genes which were not associated with the trait in either population; (ii) population-specific genes that were related to the trait in the EAS or EUR population; (iii) population-common genes that were shared across the two populations. To understand the characteristics of these genes in distinct groups, we used several conservation scores to examine the extent to which a particular gene varied across populations, which included phyloP score [72], phastCons score [73], and dN/dS ratio [74]. Specifically, higher phyloP or phastCons score indicates more conservativeness, while smaller dN/dS ratio represents higher conservativeness. We obtained these scores from [75], and compared the average scores across all genes of these traits in the three groups described above using the Friedman *F* test method.

## Results

### Estimated false discovery rate and statistical power

**Fig. 1:**
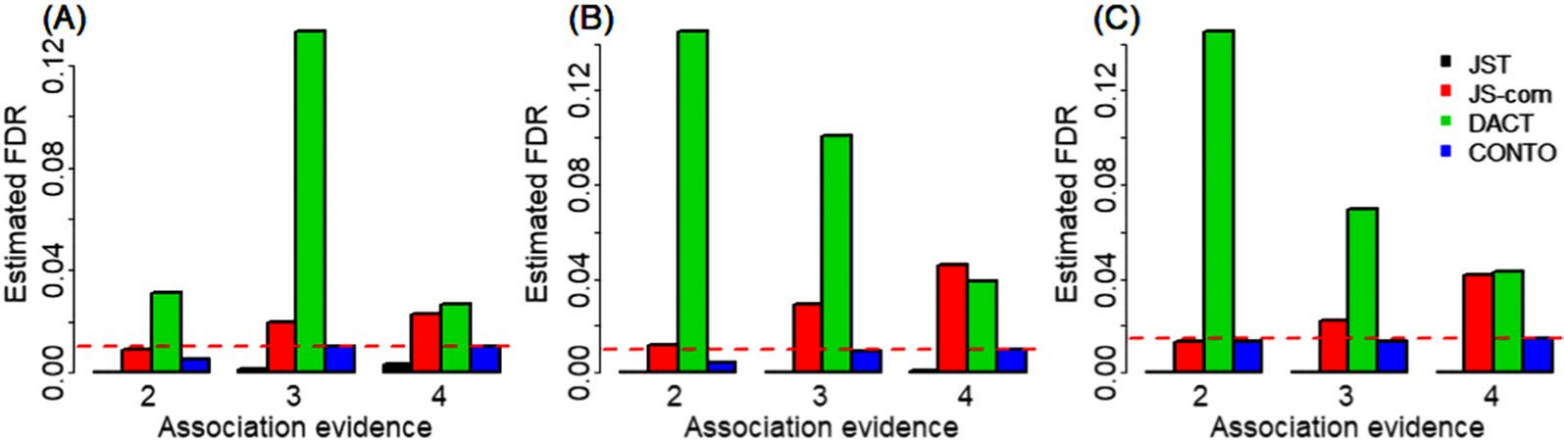
Estimated false discovery rate under the simulation settings: **A** λ_00_ = 0.40, λ_10_ = 0.20, λ_01_ = 0.20, and λ_11_ = 0.2; **B** λ_00_ = 0.80, λ_10_ = 0.05, λ_01_ = 0.05, and λ_11_ = 0.10, and **C** λ_00_ = 0.90, λ_10_ = 0.01, λ_01_ = 0.01, and λ_11_ = 0.08. Here, the number of genes was set to 15000, and the false discovery rate was calculated as the proportion of non-overlapped associated genes among all identified ones

We first assessed whether these methods could correctly control FDR at a given level. Here, we primarily focused on the results obtained under the setting that the number of genes was set to 15000 (Fig. 1). First, it is shown that JS-com could lead to inflated control of FDR under the polygenic and less overlapped case regardless of the magnitude of association signals (Fig. 1A). JS-com could maintain an efficient FDR control if the genetic architecture was less polygenic or sparse, but moderately or highly overlapped, especially when the association evidence was weak (e.g., *τ*_10_ = *τ*_01_ = *τ*_11_ = 2) (Fig. 1B–C). However, as the increase of association evidence the estimated FDR of JS-com became inflated, which was particularly evident when the trait had a less polygenic but highly overlapped genetic architecture (Fig. 1B–C). This finding was consistent with that observed in previous mediation analysis literature because the assumption of weak association signal was violated in JS-com as the increase in *τ*_10_, *τ*_01_ and *τ*_11_ [64, 65].

Second, DACT always resulted in overestimated FDR in our simulation scenarios; however, the inflation seemed to be less obvious as the genetic impact became strong if the genetic architecture of the trait was less polygenic or sparse but highly overlapped (Fig. 1B–C). Third, in contrast to DACT and JS-com, JST was overly conservative under all our simulation settings, which was in line with prior observations [44]. Fourth, CONTO effectively controlled FDR at the nominal level across our simulation cases; however, it was slightly conservative when the association evidence was relatively weak (e.g., *τ*_10_ = *τ*_01_ = *τ*_11_ = 2) under the genetic architecture which was highly polygenic but less overlapped (Fig. 1A), or less polygenic but moderately overlapped (Fig. 1B).

**Fig. 2:**
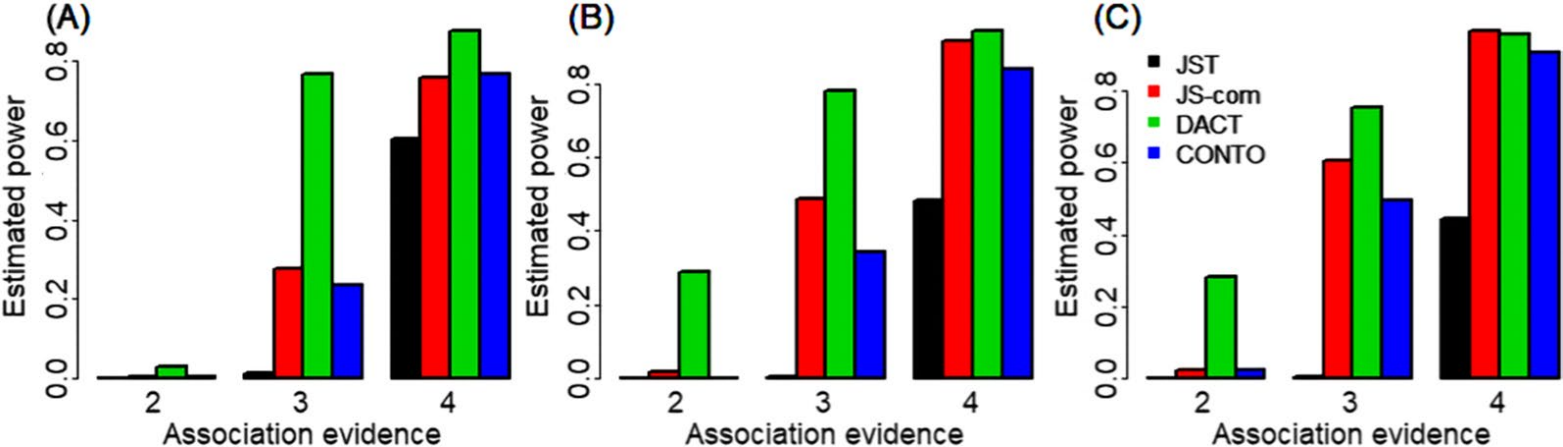
Estimated statistical power under the simulation settings: **A** λ_00_ = 0.40, λ_10_ = 0.20, λ_01_ = 0.20, and λ_11_ = 0.2; **B** λ_00_ = 0.80, λ_10_ = 0.05, λ_01_ = 0.05, and λ_11_ = 0.10, and **C** λ_00_ = 0.90, λ_10_ = 0.01, λ_01_ = 0.01, and λ_11_ = 0.08. Here, the number of genes was set to 15000, and the power was calculated as the proportion of truly overlapped associated genes among all identified ones

As shown above, because only JST and CONTO could maintain FDR at or below the given nominal level, we thus mainly considered these two methods in our following analyses. When assessing power, it was obviously observed that CONTO was consistently more powerful compared to JST across our simulation scenarios (Fig. 2). For instance, under the polygenic but less overlapped case, CONTO had a 26.6% higher power compared to JST when the association evidence was strong (τ10 = τ01 = τ11 = 4) and the number of tested genes was 15000 (Fig. 2A). The advantage of CONTO over JST became more remarkable under other two simulation scenarios (Fig. 2B–C). Finally, it was found that the similar patterns of FDR control and power behaviors were consistently observed when the number of tested genes was 10000 or 20000 (Additional file 1: Figure S1–S4).

### Associated genes discovered by MAGMA and CONTO

Based on the association results of MAGMA, we identified a set of trait-associated genes (FDR  < 0.01) shared across the EAS and EUR populations using JST and CONTO (Table 1). Consistent with the results shown in the simulation studies above, we found that CONTO had higher power and thus discovered approximately two-fold more associated genes compared to JST across all the traits, with the average number of shared associated genes increased from 109 detected by JST to 291 discovered by CONTO. Moreover, for every trait all genes identified by JST were also simultaneously detected by CONTO. With regards to CONTO, the number of detected genes related to the trait in both populations ranged from 53 for AOA to 1,544 for Height.

**Fig. 3:**
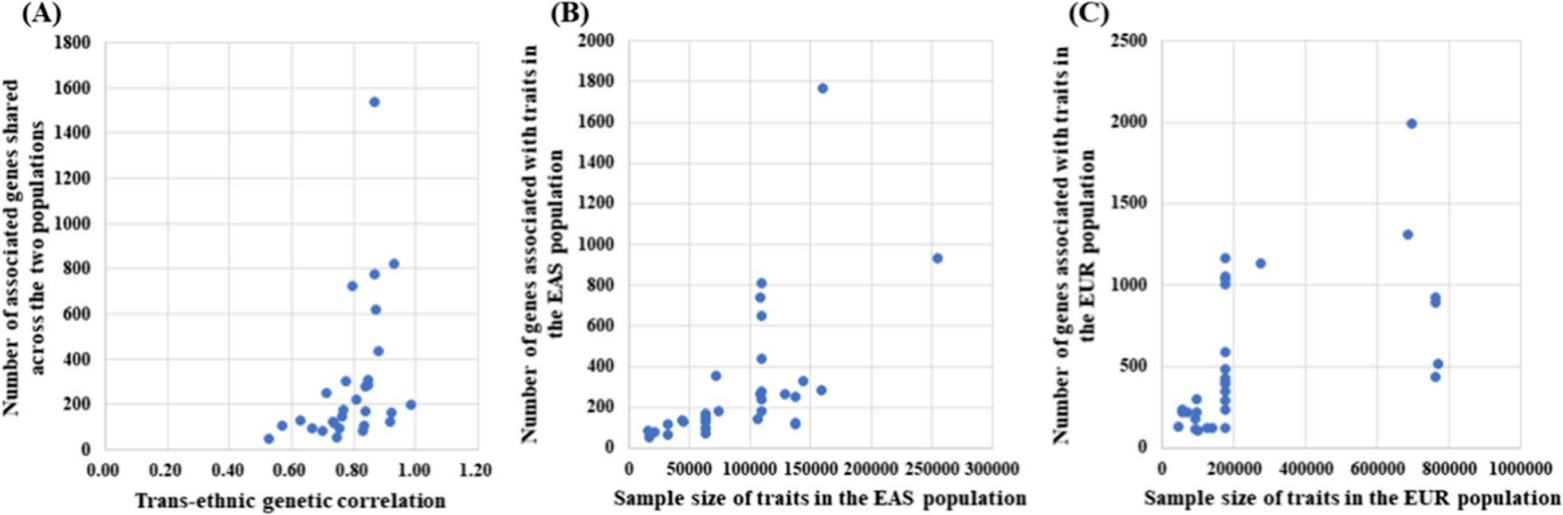
**A** Relationship between the number of population-common genes identified by CONTO and the trans-ethnic genetic correlation calculated with the popcorn method; **B** Relationship between the number of associated genes identified by MAGMA and the sample size of each trait in the EAS population; and **C** Relationship between the number of associated genes identified by MAGMA and the sample size of each trait in the EUR population

As expected, the number of common traits-associated genes was highly correlated with the trans-ethnic genetic overlap (Spearman correlation = 0.593, *P* = 8.75 × 10^−4^) (Fig. 3A). Conceptually, greater trans-ethnic genetic correlation implies higher degree of common genetic foundation underlying the trait between two diverse populations [26, 28, 42]. For example, the trans-ethnic genetic correlation was 0.93 (se = 0.04) (calculated with the popcorn method [26]) and the number of population-common associated genes was 824 for T2D; while the trans-ethnic genetic correlation was 0.53 (se = 0.11) and the number of population-common associated genes was 53 for adult-onset asthma.

Besides population-common genes, we could also identify many population-specific genes (FDR  < 0.01) (Table 1). It is evident that more population-specific genes were detected for traits in the EUR population because of higher power resulting from larger sample size (Fig. 3B–C). Consequently, the majority (an average of 92.4% across all traits) of trait-associated genes identified in the EAS population were also discovered in the EUR population, while only 56.1% of trait-associated genes identified in the EUR population were detected in the EAS population.

### Conservation of associated genes

**Fig. 4:**
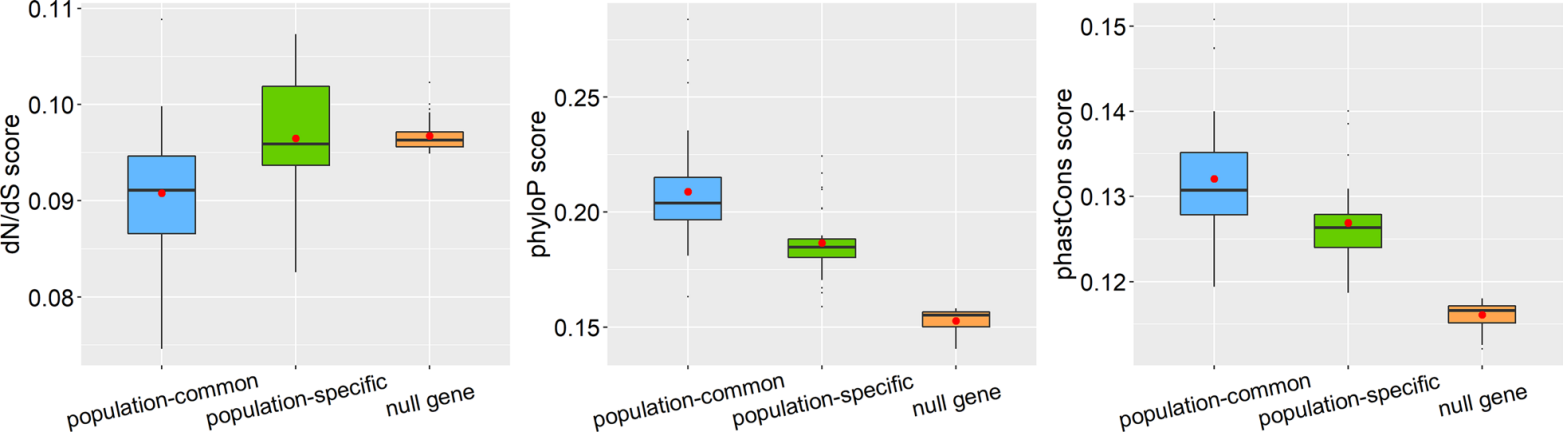
Average dN/dS ratio score **A**, phyloP score **B**, and phastCons score **C** across genes for all traits in distinct gene groups

In terms of conservation score analysis, we observed that these population-common genes were often more evolutionarily conserved compared to unique associated genes identified in a single population, which were also more evolutionarily conserved than null genes. The increased conservation pattern in population-common genes was reflected by each of the three conservation scores including dN/dS ratio (Fig. 4A), phyloP score (Fig. 4B), and phastCons score (Fig. 4C). More specifically, the average phyloP scores were 0.209, 0.188, and 0.153 for common trait-associated genes, population-specific trait-associated genes, and null genes (*P* = 1.14 × 10^−11^), respectively; the corresponding average phastCons scores were 0.132, 0.127, and 0.116 (*P* = 9.36 × 10^−14^), and the corresponding average dN/dS ratio were 0.091, 0.096, and 0.097 (*P* = 6.86 × 10^−10^).

## Discussion

As large-scale medical genomics studies have become increasingly diverse, understanding similarity and diversity of associated genes for complex diseases/traits and consequently the transferability of genetic discoveries across various populations is essential [76]. In the present study, we proposed a novel gene-centric method called CONTO to identify common associated genes shared across distinct populations by leveraging GWAS summary statistics. As a result, we detected many population-common association signals for complex diseases and traits. From a biological perspective, the existence of population-common genes is to some extent widespread because shared genetic loci are often believed to be of ancient origin and thus shared among diverse ancestral groups [77].

We also discovered a great deal of population-specific associated genes, indicating that genetic association discoveries identified in one population were not always generalized to other ancestral groups although there indeed shows substantial trans-ethnic genetic overlap underlying diseases/traits between distinct populations. These population-specific discoveries also imply the urgent requirement of including sufficient individuals from underrepresented populations in trans-ethnic GWASs so that more ethnic groups can benefit from current medical genomics researches [18, 40, 78]. The genetic diversity can be explained by the difference in clinical definitions and phenotype measurements, gene-gene and gene-environment interaction [79, 80]. As another interpretation, the genetic inconsistency across various populations might result from various sample sizes and therefore different statistical power of studies. Furthermore, we revealed that population-common genes were in general more evolutionarily conserved compared to these population-specific or null ones.

Our work distinguishes itself from previous studies in three aspects. First, unlike prior studies which often focused only on a single trait [22, 81, 82], we considered a lot of diseases/traits, which could offer more unbiased insights into the extent of trans-ethnic genetic foundation shared across different populations. Second, in contrast to prior studies which performed single-marker association analysis, we here implemented a gene-centric trans-ethnic genetic overlap identification which jointly analyzed the association of a set of SNPs with the trait. Gene-set analysis is a popular complementary strategy in association studies and often much powerful as it aggregates multiple weak, sparse association signals at the SNP level into a strong association signal at the gene level and effectively reduces the burden of multiple tests [53]. In addition, correlation among SNPs in a gene was also considered in our gene-set association analysis; consequently, the trans-ethnic difference of LD structures was naturally explained by CONTO. Third, methodologically, we considered the trans-ethnic genetic overlap identification from a perspective of composite null hypothesis testing, which effectively takes the nature of trans-ethnic genetic overlap test into account and is thus much powerful compared to competitive methods.

There are two directions that need to further explore for CONTO in the future. First, when implementing CONTO, it requires us to first generate gene-level *P* values using gene-set association analysis methods. Certainly, the used gene-set based method would play a critical role in CONTO. Intuitively, the performance of a gene-set association analysis method is determined by how well its modeling assumption matches the true genetic architecture of a group of SNPs [83, 84], which however is generally unknown a priori and varies from one gene to another. Consequently, it is difficult to choose a consistently optimal gene-set based method for all genes across the whole genome [52, 85, 86]. In the present study, we applied MAGMA to simultaneously examine the association evidence of multiple SNPs, which was in nature a variance-component based score test for multilocus genetic association studies built based on random-effects models [53, 87]. Although MAGMA might be not the optimal method for every gene, it exhibited excellent performance in statistical power compared to many existing gene-set based methods and was widely used in gene-centric integrative analysis in post-GWAS era [52–55].

Nevertheless, the use of robust and powerful gene-set based methods in CONTO is of importance. Prior studies have demonstrated that aggregating association evidence available from diverse sources is an effective strategy for boosting power; such as integrating multiple gene expression prediction models in transcriptome-wide association studies using the harmonic mean *P*-value combining method [88], and combining a group of methods into a single powerful omnibus test using the minimum *P*-value method [86] or the aggregated Cauchy combination method [89, 90]. Therefore, leveraging these similar aggregation strategies to CONTO is an interesting direction that warrants further explorations.

Second, to a great degree, CONTO should be viewed as a qualitative trans-ethnic genetic overlap identification method because it can only offer the determination whether a particular gene is associated with the trait in both populations, but is difficult to give accurate evidence regarding the consistence of trans-ethnic genetic effects across populations. Understanding the effect difference could provide more in-depth insight into the diversity and similarity of genetic architecture underlying the same trait across distinct ancestral groups. To examine the genetic influence of a gene on complex traits in GWAS, prior studies attempted to employ the polygenic risk score calculated based on SNP effect sizes and genotypes of individuals available from external reference panels [2, 30, 54, 55]. Thus, such score may be also useful for the evaluation of trans-ethnic genetic effect of a gene in CONTO.

## Conclusion

In summary, CONTO stands for a powerful method for detecting trans-ethnic genetic overlap across diverse ancestral groups; our results provide important implications on the transferability of GWAS discoveries in one population to others.

## Supplementary Information

**Additional file 1: Table S1. **Complex traits available from the European and East Asian analyzed in the present study. **Figure S1. **Estimated false discovery rate under the simulation settings: (A) λ_00_=0.40, λ_10_=0.20, λ_01_=0.20, and λ_11_=0.2; (B) λ_00_=0.80, λ_10_=0.05, λ_01_=0.05, and λ_11_=0.10, and (C) λ_00_=0.90, λ_10_=0.01, λ_01_=0.01, and λ_11_=0.08. Here, the number of genes was set to 10000, and the false discovery rate was calculated as the proportion of non-overlapped associated genes among all identified ones. **Figure S2.** Estimated statistical power under the simulation settings: (A) λ_00_=0.40, λ_10_=0.20, λ_01_=0.20, and λ_11_=0.2; (B) λ_00_=0.80, λ_10_=0.05, λ_01_=0.05, and λ_11_=0.10, and (C) λ_00_=0.90, λ_10_=0.01, λ_01_=0.01, and λ_11_=0.08. Here, the number of genes was set to 10000, and the power was calculated as the proportion of truly overlapped associated genes among all identified ones. **Figure S3.** Estimated false discovery rate under the simulation settings: (A) λ_00_=0.40, λ_10_=0.20, λ_01_=0.20, and λ_11_=0.2; (B) λ_00_=0.80, λ_10_=0.05, λ_01_=0.05, and λ_11_=0.10, and (C) λ_00_=0.90, λ_10_=0.01, λ_01_=0.01, and λ_11_=0.08. Here, the number of genes was set to 20000, and the false discovery rate was calculated as the proportion of non-overlapped associated genes among all identified ones. **Figure S4.** Estimated statistical power under the simulation settings: (A) λ_00_=0.40, λ_10_=0.20, λ_01_=0.20, and λ_11_=0.2; (B) λ_00_=0.80, λ_10_=0.05, λ_01_=0.05, and λ_11_=0.10, and (C) λ_00_=0.90, λ_10_=0.01, λ_01_=0.01, and λ_11_=0.08. Here, the number of genes was set to 20000, and the power was calculated as the proportion of truly overlapped associated genes among all identified ones.

## Data Availability

All data generated or analyzed during this study are included in this published article and its supplementary information file.

## Abbreviations

CONTO: Composite null hypothesis test for trans-ethnic genetic overlap
GWASs: Genome-wide association studies
SNPs: Single nucleotide polymorphisms
EUR: European
JST: Joint significance test
EAS: East Asian
FDR: False discovery rate
MVN: Multivariate normal
JT-comp: Joint significance composite-null test
DACT: Divide-aggregate composite-null test
