# Supplementary material for: Detecting associated genes for complex traits shared across East Asian and European populations under the framework of composite null hypothesis testing
Source: J Transl Med. 2022 Sep 23;20:424. doi: 10.1186/s12967-022-03637-8 (PMC9503281; doi:10.1186/s12967-022-03637-8)
Supplement: Supplementary file 1 — Additional file 1: Table S1. Complex traits available from the European and East Asian analyzed in the present study. Figure S1. Estimated false discovery rate under the simulation settings: (A) λ00=0.40, λ10=0.20, λ01=0.20, and λ11=0.2; (B) λ00=0.80, λ10=0.05, λ01=0.05, and λ11=0.10, and (C) λ00=0.90, λ10=0.01, λ01=0.01, and λ11=0.08. Here, the number of genes was set to 10000, and the false discovery rate was calculated as the proportion of non-overlapped associated genes among all identified ones. Figure S2. Estimated statistical power under the simulation settings: (A) λ00=0.40, λ10=0.20, λ01=0.20, and λ11=0.2; (B) λ00=0.80, λ10=0.05, λ01=0.05, and λ11=0.10, and (C) λ00=0.90, λ10=0.01, λ01=0.01, and λ11=0.08. Here, the number of genes was set to 10000, and the power was calculated as the proportion of truly overlapped associated genes among all identified ones. Figure S3. Estimated false discovery rate under the simulation settings: (A) λ00=0.40, λ10=0.20, λ01=0.20, and λ11=0.2; (B) λ00=0.80, λ10=0.05, λ01=0.05, and λ11=0.10, and (C) λ00=0.90, λ10=0.01, λ01=0.01, and λ11=0.08. Here, the number of genes was set to 20000, and the false discovery rate was calculated as the proportion of non-overlapped associated genes among all identified ones. Figure S4. Estimated statistical power under the simulation settings: (A) λ00=0.40, λ10=0.20, λ01=0.20, and λ11=0.2; (B) λ00=0.80, λ10=0.05, λ01=0.05, and λ11=0.10, and (C) λ00=0.90, λ10=0.01, λ01=0.01, and λ11=0.08. Here, the number of genes was set to 20000, and the power was calculated as the proportion of truly overlapped associated genes among all identified ones. [file 12967_2022_3637_MOESM1_ESM.docx]

**Supplement File**

## Table S1. Complex traits available from the European and East Asian analyzed in the present study

| no | trait | East Asian | |  | European | |
| --- | --- | --- | --- | --- | --- | --- |
|  |  | *k*_1_ | Ref |  | *k*_2_ | Ref |
| binary trait | |  |  |  |  |  |
| 1 | SCZ | 8,962,953 | [[1](#_ENREF_1)] |  | 9,387,791 | [[2](#_ENREF_2)] |
| 2 | RA | 5,465,181 | [[3](#_ENREF_3)] |  | 4,506,924 | [[3](#_ENREF_3)] |
| 3 | T2D | 12,138,493 | [[4](#_ENREF_4)] |  | 8,768,343 | [[5](#_ENREF_5)] |
| 4 | COA | 8,614,703 | [[6](#_ENREF_6)] |  | 6,530,817 | [[7](#_ENREF_7)] |
| 5 | AOA | 8,579,847 | [[6](#_ENREF_6)] |  | 6,530,817 | [[7](#_ENREF_7)] |
| 6 | PCA | 8,075,653 | [[6](#_ENREF_6)] |  | 6,530,649 | [[8](#_ENREF_8)] |
| continuous trait | | | | | | |
| 1 | BMI | 2,336,155 | [[9](#_ENREF_9)] |  | 5,834,937 | [[10](#_ENREF_10)] |
| 2 | height | 2,254,569 | [[11](#_ENREF_11)] |  | 7,171,865 | [[10](#_ENREF_10)] |
| 3 | DBP | 7,080,713 | [[11](#_ENREF_11)] |  | 5,834,937 | [[12](#_ENREF_12)] |
| 4 | SBP | 7,009,162 | [[11](#_ENREF_11)] |  | 5,834,937 | [[12](#_ENREF_12)] |
| 5 | PP | 7,009,859 | [[11](#_ENREF_11)] |  | 5,834,937 | [[12](#_ENREF_12)] |
| 6 | HDL | 2,253,836 | [[11](#_ENREF_11)] |  | 5,834,937 | [[13](#_ENREF_13)] |
| 7 | LDL | 2,245,087 | [[11](#_ENREF_11)] |  | 5,834,937 | [[13](#_ENREF_13)] |
| 8 | TC | 2,252,665 | [[11](#_ENREF_11)] |  | 5,834,937 | [[13](#_ENREF_13)] |
| 9 | TG | 2,246,337 | [[11](#_ENREF_11)] |  | 5,834,937 | [[13](#_ENREF_13)] |
| 10 | HbA1c | 2,562,190 | [[11](#_ENREF_11)] |  | 5,834,937 | [[14](#_ENREF_14)] |
| 11 | eGFR | 8,450,424 | [[11](#_ENREF_11)] |  | 5,834,937 | [[15](#_ENREF_15)] |
| 12 | ANM | 2,369,171 | [[11](#_ENREF_11)] |  | 7,970,962 | [[16](#_ENREF_16)] |
| 13 | PLT | 8,399,937 | [[11](#_ENREF_11)] |  | 5,834,937 | [[17](#_ENREF_17)] |
| 14 | RBC | 8,401,330 | [[11](#_ENREF_11)] |  | 5,834,937 | [[17](#_ENREF_17)] |
| 15 | MCV | 8,402,224 | [[11](#_ENREF_11)] |  | 5,834,937 | [[17](#_ENREF_17)] |
| 16 | HCT | 8,401,908 | [[11](#_ENREF_11)] |  | 5,834,937 | [[17](#_ENREF_17)] |
| 17 | MCH | 8,400,817 | [[11](#_ENREF_11)] |  | 5,834,937 | [[17](#_ENREF_17)] |
| 18 | MCHC | 8,403,148 | [[11](#_ENREF_11)] |  | 5,834,937 | [[17](#_ENREF_17)] |
| 19 | HGB | 8,401,191 | [[11](#_ENREF_11)] |  | 5,834,937 | [[17](#_ENREF_17)] |
| 20 | MONO | 8,399,762 | [[11](#_ENREF_11)] |  | 5,834,937 | [[17](#_ENREF_17)] |
| 21 | NEUT | 8,400,293 | [[11](#_ENREF_11)] |  | 5,834,937 | [[17](#_ENREF_17)] |
| 22 | EO | 8,400,560 | [[11](#_ENREF_11)] |  | 5,834,937 | [[17](#_ENREF_17)] |
| 23 | BASO | 8,399,810 | [[11](#_ENREF_11)] |  | 5,834,937 | [[17](#_ENREF_17)] |
| 24 | LYMPH | 8,399,656 | [[11](#_ENREF_11)] |  | 5,834,937 | [[17](#_ENREF_17)] |
| 25 | WBC | 8,401,104 | [[11](#_ENREF_11)] |  | 5,834,937 | [[17](#_ENREF_17)] |

Note: *k*_1_ and *k*_2_ are the number of SNPs in the original EAS and EUR GWASs. SCZ: schizophrenia; RA: rheumatoid arthritis; T2D: type 2 diabetes; COA: childhood-onset asthma; AOA: adult-onset asthma; PCA: prostate cancer; BMI: body mass index; DBP: diastolic blood pressure; SBP: systolic blood pressure; PP: pulse pressure; HDL: high density lipoprotein cholesterol; LDL: low density lipoprotein cholesterol; TC: total cholesterol; TG: triglyceride; HbA1c: hemoglobin A1c; eGFR: estimated glomerular filtration rate; ANM: age at natural (non-surgical) menopause; PLT: platelet count; RBC: red blood cell count; MVC: mean corpuscular volume; HCT: hematocrit; MCH: mean corpuscular hemoglobin; MCHC: mean corpuscular hemoglobin concentration; HGB: hemoglobin concentration; MONO: monocyte count; NEUT: neutrophil count; EO: eosinophil count; BASO: basophil count; LYMPH: lymphocyte count; WBC: white blood cell count.


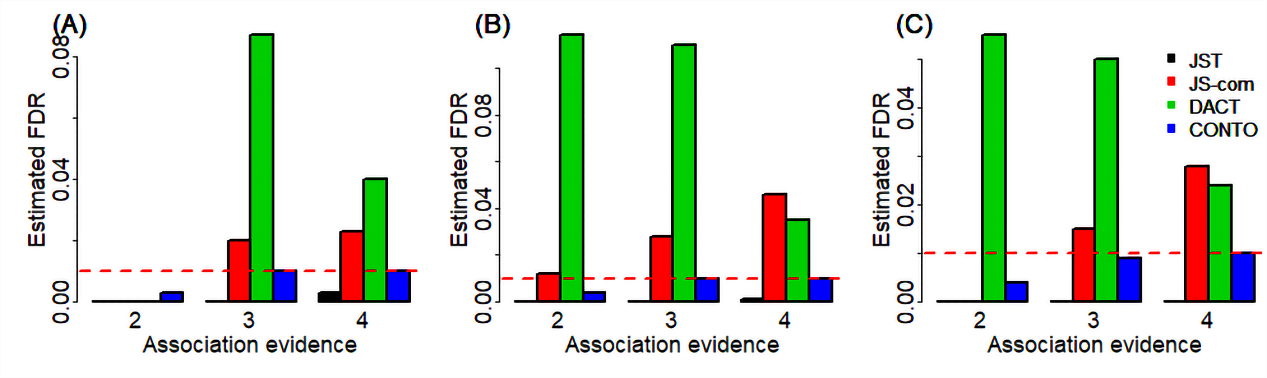


Figure S1. Estimated false discovery rate under the simulation settings: (A) λ_00_=0.40, λ_10_=0.20, λ_01_=0.20, and λ_11_=0.2; (B) λ_00_=0.80, λ_10_=0.05, λ_01_=0.05, and λ_11_=0.10, and (C) λ_00_=0.90, λ_10_=0.01, λ_01_=0.01, and λ_11_=0.08. Here, the number of genes was set to 10000, and the false discovery rate was calculated as the proportion of non-overlapped associated genes among all identified ones.


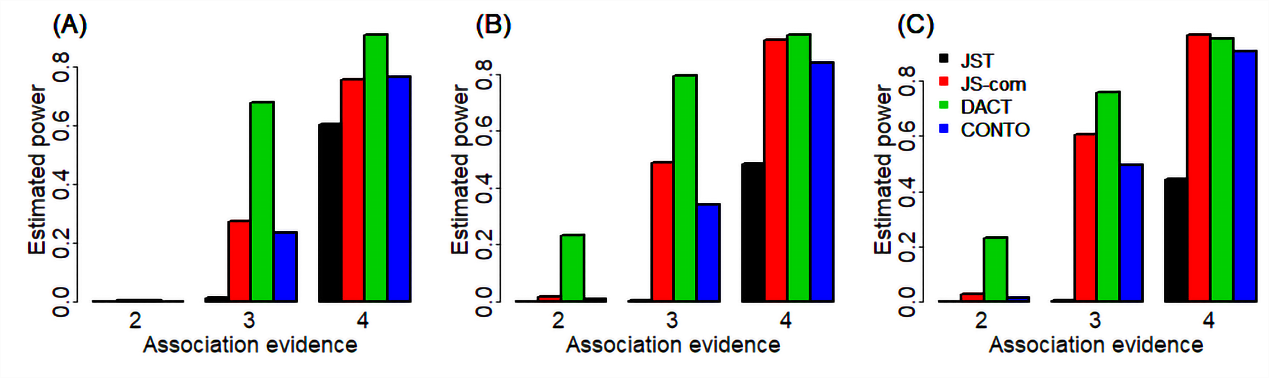


Figure S2. Estimated statistical power under the simulation settings: (A) λ_00_=0.40, λ_10_=0.20, λ_01_=0.20, and λ_11_=0.2; (B) λ_00_=0.80, λ_10_=0.05, λ_01_=0.05, and λ_11_=0.10, and (C) λ_00_=0.90, λ_10_=0.01, λ_01_=0.01, and λ_11_=0.08. Here, the number of genes was set to 10000, and the power was calculated as the proportion of truly overlapped associated genes among all identified ones.


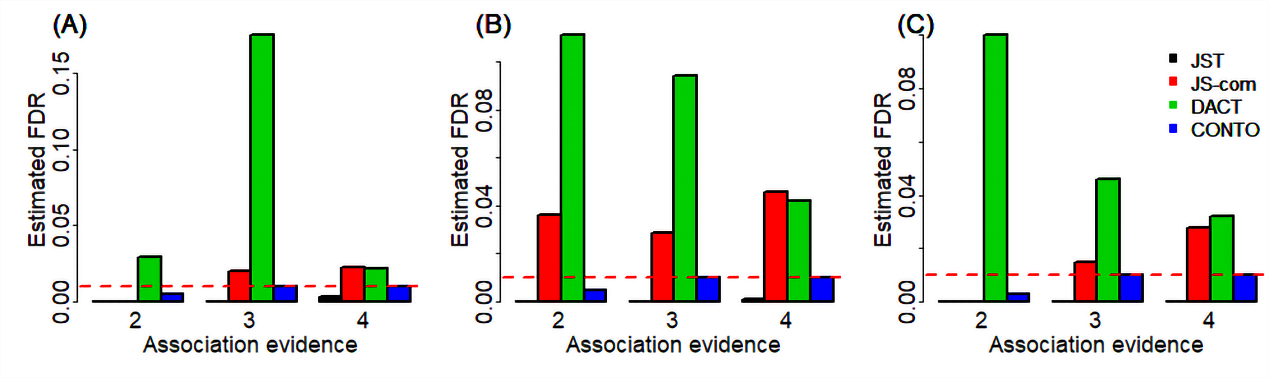


Figure S3. Estimated false discovery rate under the simulation settings: (A) λ_00_=0.40, λ_10_=0.20, λ_01_=0.20, and λ_11_=0.2; (B) λ_00_=0.80, λ_10_=0.05, λ_01_=0.05, and λ_11_=0.10, and (C) λ_00_=0.90, λ_10_=0.01, λ_01_=0.01, and λ_11_=0.08. Here, the number of genes was set to 20000, and the false discovery rate was calculated as the proportion of non-overlapped associated genes among all identified ones.


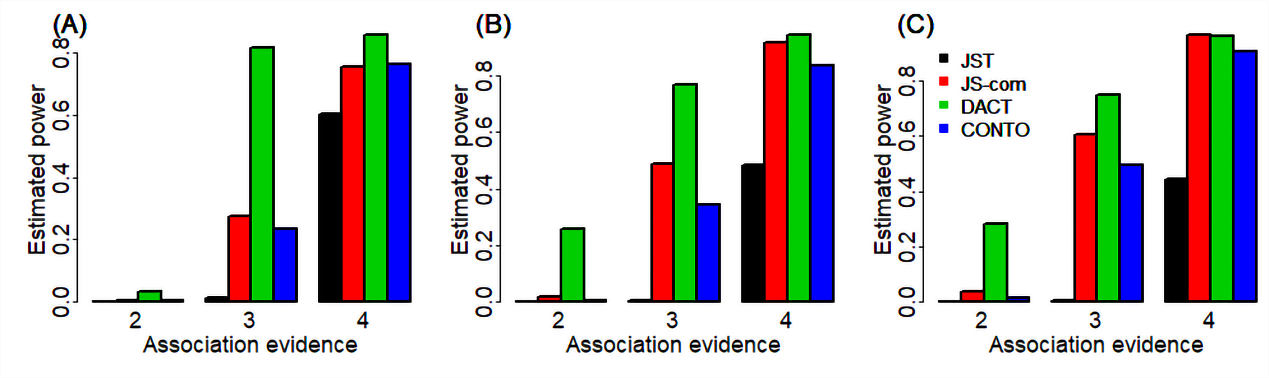


Figure S4. Estimated statistical power under the simulation settings: (A) λ_00_=0.40, λ_10_=0.20, λ_01_=0.20, and λ_11_=0.2; (B) λ_00_=0.80, λ_10_=0.05, λ_01_=0.05, and λ_11_=0.10, and (C) λ_00_=0.90, λ_10_=0.01, λ_01_=0.01, and λ_11_=0.08. Here, the number of genes was set to 20000, and the power was calculated as the proportion of truly overlapped associated genes among all identified ones.

### Composite trans-ethnic genetic overlap test

Our primary objective is to examine the extent to which a particular trait-associated gene identified in one population is also significant in another population. Form a statistical perspective, this trans-ethnic genetic overlap can be defined in terms of distinct types of summary statistics shown above. For example, based on effect sizes the alternative hypothesis can be expressed as *H*_11_: *β*_1_≠0 and *β*_2_≠0; or alternatively, shared genetic loci across ancestral groups implies that both |*Z*_1_| and |*Z*_2_| are larger than a pre-assigned threshold value, or that both *P*_1_ and *P*_2_ are less than a given significance level. This alternative hypothesis corresponds to three composite null sub-hypotheses. (i) *H*_00_: the gene is not associated with the trait in neither population; (ii) *H*_10_: the gene is associated with the trait in the first population but not the second; (iii) *H*_01_: the gene is associated with the trait in the second population but not the first. Formally, if defining the hypothesis test according to *P* values, we have

where *α* is the given significance level. Under this framework we intend to identify shared trait-associated genes in both populations from the viewpoint of composite null hypothesis testing.

### Composite-null test methods for trans-ethnic genetic loci

#### Joint significance test

First, to examine the composite null hypothesis shown in , it is very natural to take the maximum value of *P*_1_ and *P*_2_

$$P_{\text{max}} = \text{max}(P_{1}, P_{2})$$

as the measurement of the existence of trans-ethnic genetic overlap for a particular gene. This method is thus referred to as the maximum *P*-value method, which is also known as the joint significance test (JST) [[18](#_ENREF_18)]. Afterwards, JST deems the gene to be commonly significant at a level *α* across populations if and only if *P*_max_ is significant at the level *α* (i.e., *P*_max_<*α*). Essentially, JST is a type of intersection-union test and is a level-*α* test with type I error guaranteed to be at most *α* [[19-21](#_ENREF_19)]. However, because of failing to consider the composite null nature of the trans-ethnic genetic overlap test, JST is often much conservative and the resulting type I error equals *α* only under certain regularity conditions [[22](#_ENREF_22), [23](#_ENREF_23)].

#### Joint significance composite-null test (JT-comp)

Unlike prior methods, JT-comp depends on the two *z*-scores *Z*_1_ and *Z*_2_ [[24](#_ENREF_24)], and derives the null distribution of their product by carefully examining the two *z*-statistics under the three sub-null scenarios. Specifically, under *H*_01_, *Z*_1_ is assumed to follow a standard normal distribution while *Z*_2_ is assumed to follow a normal distribution *N*(*μ*_1_, 1) with mean *μ*_1_ characterized by *β*_2_. Under *H*_10_, *Z*_2_ is assumed to follow a standard normal distribution while *Z*_1_ follows a normal distribution *N*(*μ*_1_, 1) with mean *μ*_1_ characterized by *β*_1_. Under *H*_00_, both *Z*_1_ and *Z*_2_ are assumed to follow the standard normal distribution. Therefore, one can compute the *P* value for the product of *Z*_1_ and *Z*_2_ under *H*_00_ directly, whereas calculating the *P* value under either *H*_01_ or *H*_10_ requires the knowledge of *μ*_1_ and *μ*_2_. JT-comp overcomes this difficulty by assigning priors for them *μ*_1_~*N*(0, *τ*_1_) and *μ*_2_~*N*(0, *τ*_2_); then, $z_{1}z_{2}/\sqrt{1+\tau_{2}}$ and $z_{1}z_{2}/\sqrt{1+\tau_{1}}$ become a product of two independent standard normal distributions under *H*_01_ or *H*_10_ given *τ*_1_ and *τ*_2_.

To further circumvent the difficulty of estimating unknown *τ*_1_ and *τ*_2_, JT-comp assumes the two parameters are relatively small and that the association signals are sparse [[24](#_ENREF_24)]. With these conditions, JT-comp approximately computes the final *P* value for each gene through

$$P_{\text{JT-comp}} \approx F\left(Z_{1}\frac{Z_{2}}{\sqrt{\mathrm{var}(Z_{2})}}\right) + F\left(\frac{Z_{1}}{\sqrt{\mathrm{var}(Z_{1})}}Z_{2}\right) \text{-} F\left(Z_{1}Z_{2}\right)$$

where $F\left( z \right)=\int_{|z|}^{\infty} f(x)dx$ is the right-sided tail probability of a normal product distribution calculated at point *z*, with *f*(*x*)=*K*_0_(*x*)/*π* (-∞<*x*<∞) the probability density function of the normal product distribution and *K*_0_(*x*) being the modified Bessel function of the second kind with order 0, and var(*Z*_1_) and var(*Z*_2_) are the sample variance of *Z*_1_ and *Z*_2_, respectively.

#### Divide-aggregate composite-null test (DACT)

DACT is another composite-null test method proposed recently in high-dimensional data analysis [[25](#_ENREF_25)]. Different from JT-comp, DACT relies on *P* values, and constructs a modified test statistic by directly estimating the proportions of the three sub-null hypotheses across all genetic overlap tests. Intuitively, because the gene is associated the trait in the second population under *H*_01_ (i.e., *β*_2_≠0), we only need to examine *H*_01_: *β*_1_=0 and employ *P*_1_ for assessing the existence of the association in the first population. In the same way, because the gene is associated the trait in the first population under *H*_10_ (i.e., *β*_1_≠0), we only need to evaluate *H*_10_: *β*_2_=0 and apply *P*_2_ for evaluating the presence of the association in the second population. In addition, the maximum *P* value of the two, *P*_max_, follows Beta(2, 1) under *H*_00_; as a result, $P_{\max}^{2}$, rather than *P*_max_ as done in JST, follows a uniform distribution [[25](#_ENREF_25)]. Taking these together, DACT generates the *P* value as a weighted summation of *P* values under the three sub-null hypotheses

$$P_{\text{DACT}} = w_{01}P_{1} + w_{10}P_{2} + w_{00}P_{\text{max}}^{2}$$

where the weights are given as

$$w_{01} \propto(1 \text{-} \text{λ}_{01})\text{λ}_{10}, w_{10} \propto(1 \text{-} \text{λ}_{01})\text{λ}_{01}, w_{00} \propto(1 \text{-} \text{λ}_{10})(1 \text{-} \text{λ}_{01})$$

DACT estimates λ_10_ and λ_01_ using novel methods that have been well-established in prior FDR work, such as Efron’s approach [[26](#_ENREF_26)] using either the central matching method [[27](#_ENREF_27)] or the empirical characteristic function and Fourier analysis [[28](#_ENREF_28)].

#### Composite null hypothesis test for trans-ethnic genetic overlap (CONTO)

CONTO follows the similar concept with JT-comp and DACT and aims to directly build the null distribution of *P*_max_ to correct for the conservative type I error control of JST. Specifically, CONTO estimates the proportions of the three sub-null hypotheses and fits a mixture null distribution for *P*_max_ through [[29](#_ENREF_29)]

$$\begin{matrix} \Pr(P_{\text{max}} \leq\alpha|H_{0}) = \Pr(P_{1} \leq\alpha|H_{01})\Pr(P_{2} \leq\alpha|H_{01})\Pr(H_{01}) \\ + \Pr(P_{1} \leq\alpha|H_{10})\Pr(P_{2} \leq\alpha|H_{10})\Pr(H_{10}) \\ + \Pr(P_{1} \leq\alpha|H_{00})\Pr(P_{2} \leq\alpha|H_{00})\Pr(H_{00}) \\ = \text{λ}_{01}p_{01}\alpha+ \text{λ}_{10}p_{10}\alpha+ \text{λ}_{00}\alpha^{2} \\ p_{01} = \Pr(P_{2} \leq\alpha|H_{01}) \\ p_{10} = \Pr(P_{1} \leq\alpha|H_{10}) \end{matrix}$$

where *α* is a given cut-off value for significance evaluation; *p*_01_ is the power of rejecting *β*_2_=0 under *H*_01_; and *p*_10_ is the power of rejecting *β*_1_=0 under *H*_10_, both of which is estimated via the Grenander method [[30](#_ENREF_30)].

The proportion parameters required in CONTO are also estimated with the same methods as used in DACT using the method given in [[31-33](#_ENREF_31)]. Specifically, let λ_0+_ be the null proportion of *P*_1_*_j_* and λ_+0_ be the null proportion of *P*_2_*_j_*; then λ_0+_ and λ_+0_ can be estimated by

$$\begin{matrix} \hat{\text{λ}}_{0+}(c_{1}) = \frac{1}{L(1 - c_{1})}\text{\#}\left\{P_{1j} > c_{1}\right\} \\ \hat{\text{λ}}_{+0}(c_{2}) = \frac{1}{L(1 - c_{2})}\text{\#}\left\{P_{2j} > c_{2}\right\} \end{matrix}$$

where *L* is the total number of genes under investigation, and *c*_1_ and *c*_2_ are two pre-selected tuning parameters to determine whether an observed *P* value comes from the null case. Additionally, λ_00_ is calculated as

$$\hat{\text{λ}}_{00}\text{(}c_{1}, c_{2}) = \frac{1}{L(1 - c_{1})(1 - c_{2})}\text{\#}\left\{P_{1j} > c_{1}, P_{2j} > c_{2}\right\}$$

For simplicity and conservativeness, in the present study we use *c*_1_=*c*_2_=0.5 following previous work [[29](#_ENREF_29)]. Finally, we have

$$\hat{\text{λ}}_{01}(c_{1}) = \hat{\text{λ}}_{0+}(c_{1}) - \hat{\text{λ}}_{00}\text{(}c_{1}, c_{2}), \hat{\text{λ}}_{10}(c_{2}) = \hat{\text{λ}}_{+0}(c_{2}) - \hat{\text{λ}}_{00}\text{(}c_{1}, c_{2})$$

## References

1. Lam M, Chen C-Y, Li Z, Martin AR, Bryois J, Ma X, Gaspar H, Ikeda M, Benyamin B, Brown BC, et al: **Comparative genetic architectures of schizophrenia in East Asian and European populations.** *Nat Genet* 2019, **51:**1670-1678.

2. Ripke S, Neale BM, Corvin A, Walters JTR, Farh K-H, Holmans PA, Lee P, Bulik-Sullivan B, Collier DA, Huang H, et al: **Biological insights from 108 schizophrenia-associated genetic loci.** *Nature* 2014, **511:**421-427.

3. Okada Y, Wu D, Trynka G, Raj T, Terao C, Ikari K, Kochi Y, Ohmura K, Suzuki A, Yoshida S: **Genetics of rheumatoid arthritis contributes to biology and drug discovery.** *Nature* 2014, **506:**376-381.

4. Spracklen CN, Horikoshi M, Kim YJ, Lin K, Bragg F, Moon S, Suzuki K, Tam CHT, Tabara Y, Kwak S-H, et al: **Identification of type 2 diabetes loci in 433,540 East Asian individuals.** *Nature* 2020.

5. Mahajan A, Taliun D, Thurner M, Robertson NR, Torres JM, Rayner NW, Payne AJ, Steinthorsdottir V, Scott RA, Grarup N, et al: **Fine-mapping type 2 diabetes loci to single-variant resolution using high-density imputation and islet-specific epigenome maps.** *Nat Genet* 2018, **50:**1505-1513.

6. Ishigaki K, Akiyama M, Kanai M, Takahashi A, Kawakami E, Sugishita H, Sakaue S, Matoba N, Low S-K, Okada Y, et al: **Large-scale genome-wide association study in a Japanese population identifies novel susceptibility loci across different diseases.** *Nat Genet* 2020.

7. Ferreira MAR, Mathur R, Vonk JM, Szwajda A, Brumpton B, Granell R, Brew BK, Ullemar V, Lu Y, Jiang Y, et al: **Genetic Architectures of Childhood- and Adult-Onset Asthma Are Partly Distinct.** *Am J Hum Genet* 2019, **104:**665-684.

8. Schumacher FR, Al Olama AA, Berndt SI, Benlloch S, Ahmed M, Saunders EJ, Dadaev T, Leongamornlert D, Anokian E, Cieza-Borrella C, et al: **Association analyses of more than 140,000 men identify 63 new prostate cancer susceptibility loci.** *Nat Genet* 2018, **50:**928-936.

9. Akiyama M, Okada Y, Kanai M, Takahashi A, Momozawa Y, Ikeda M, Iwata N, Ikegawa S, Hirata M, Matsuda K, et al: **Genome-wide association study identifies 112 new loci for body mass index in the Japanese population.** *Nat Genet* 2017, **49:**1458-1467.

10. Yengo L, Sidorenko J, Kemper KE, Zheng Z, Wood AR, Weedon MN, Frayling TM, Hirschhorn J, Yang J, Visscher PM, the GC: **Meta-analysis of genome-wide association studies for height and body mass index in ~700000 individuals of European ancestry.** *Hum Mol Genet* 2018, **27:**3641-3649.

11. Kanai M, Akiyama M, Takahashi A, Matoba N, Momozawa Y, Ikeda M, Iwata N, Ikegawa S, Hirata M, Matsuda K, et al: **Genetic analysis of quantitative traits in the Japanese population links cell types to complex human diseases.** *Nat Genet* 2018, **50:**390-400.

12. Evangelou E, Warren HR, Mosen-Ansorena D, Mifsud B, Pazoki R, Gao H, Ntritsos G, Dimou N, Cabrera CP, Karaman I, et al: **Genetic analysis of over 1 million people identifies 535 new loci associated with blood pressure traits.** *Nat Genet* 2018, **50:**1412-1425.

13. Willer CJ, Schmidt EM, Sengupta S, Peloso GM, Gustafsson S, Kanoni S, Ganna A, Chen J, Buchkovich ML, Mora S, et al: **Discovery and refinement of loci associated with lipid levels.** *Nat Genet* 2013, **45:**1274-1283.

14. Wheeler E, Leong A, Liu C-T, Hivert M-F, Strawbridge RJ, Podmore C, Li M, Yao J, Sim X, Hong J, et al: **Impact of common genetic determinants of Hemoglobin A1c on type 2 diabetes risk and diagnosis in ancestrally diverse populations: A transethnic genome-wide meta-analysis.** *PLoS Med* 2017, **14:**e1002383.

15. Wuttke M, Li Y, Li M, Sieber KB, Feitosa MF, Gorski M, Tin A, Wang L, Chu AY, Hoppmann A, et al: **A catalog of genetic loci associated with kidney function from analyses of a million individuals.** *Nat Genet* 2019, **51:**957-972.

16. Day FR, Ruth KS, Thompson DJ, Lunetta KL, Pervjakova N, Chasman DI, Stolk L, Finucane HK, Sulem P, Bulik-Sullivan B, et al: **Large-scale genomic analyses link reproductive aging to hypothalamic signaling, breast cancer susceptibility and BRCA1-mediated DNA repair.** *Nat Genet* 2015, **47:**1294-1303.

17. Astle WJ, Elding H, Jiang T, Allen D, Ruklisa D, Mann AL, Mead D, Bouman H, Riveros-Mckay F, Kostadima MA, et al: **The Allelic Landscape of Human Blood Cell Trait Variation and Links to Common Complex Disease.** *Cell* 2016, **167:**1415-1429.

18. Barfield R, Shen J, Just AC, Vokonas PS, Schwartz J, Baccarelli AA, VanderWeele TJ, Lin X: **Testing for the indirect effect under the null for genome-wide mediation analyses.** *Genet Epidemiol* 2017, **41:**824-833.

19. Berger RL: **Multiparameter Hypothesis Testing and Acceptance Sampling.** *Technometrics* 1982, **24:**295-300.

20. Berger RL, Hsu JC: **Bioequivalence trials, intersection-union tests and equivalence confidence sets.** *Stat Sci* 1996, **11:**283-319.

21. Berger RL: **Likelihood Ratio Tests and Intersection-Union Tests.** In *Advances in Statistical Decision Theory and Applications.* Edited by Panchapakesan S, Balakrishnan N. Boston, MA: Birkhäuser Boston; 1997: 225-237

22. Zeng P, Shao Z, Zhou X: **Statistical methods for mediation analysis in the era of high-throughput genomics: current successes and future challenges.** *Computational and Structural Biotechnology Journal* 2021, **19:**3209-3224.

23. Shao Z, Wang T, Zhang M, Jiang Z, Huang S, Zeng P: **IUSMMT: Survival mediation analysis of gene expression with multiple DNA methylation exposures and its application to cancers of TCGA.** *PLoS Comput Biol* 2021, **17:**e1009250.

24. Huang Y-T: **Genome-wide analyses of sparse mediation effects under composite null hypotheses.** *The Annals of Applied Statistics* 2019, **13:**60-84.

25. Liu Z, Shen J, Barfield R, Schwartz J, Baccarelli AA, Lin X: **Large-Scale Hypothesis Testing for Causal Mediation Effects with Applications in Genome-wide Epigenetic Studies.** *J Am Stat Assoc* 2021**:**1-39.

26. Efron B: **Large-Scale Simultaneous Hypothesis Testing.** *J Am Stat Assoc* 2004, **99:**96-104.

27. Efron B: **Size, power and false discovery rates.** *Ann Stat* 2007, **35:**1351-1377.

28. Jin J, Cai TT: **Estimating the Null and the Proportion of Nonnull Effects in Large-Scale Multiple Comparisons.** *J Am Stat Assoc* 2007, **102:**495-506.

29. Dai JY, Stanford JL, LeBlanc M: **A Multiple-Testing Procedure for High-Dimensional Mediation Hypotheses.** *J Am Stat Assoc* 2020**:**1-16.

30. Langaas M, Lindqvist BH, Ferkingstad E: **Estimating the proportion of true null hypotheses, with application to DNA microarray data.** *J R Stat Soc Ser B* 2005, **67:**555-572.

31. Storey J: **A direct approach to false discovery rates.** *J R Stat Soc Ser B* 2002, **64:**479 - 498.

32. Storey J: **The positive false discovery rate: a Bayesian interpretation and the q-value.** *Ann Stat* 2003, **31:**2013-2035.

33. Storey J, Tibshirani R: **Statistical significance for genomewide studies.** *Proc Natl Acad Sci U S A* 2003, **100:**9440-9445.
